# Recorded time periods of bispectral index values equal to zero predict neurological outcome after out-of-hospital cardiac arrest

**DOI:** 10.1186/s13054-017-1806-y

**Published:** 2017-08-22

**Authors:** Ward Eertmans, Cornelia Genbrugge, Gilles Haesevoets, Jo Dens, Willem Boer, Frank Jans, Cathy De Deyne

**Affiliations:** 1 Department of Medicine and Life Sciences, Hasselt University, Diepenbeek, Belgium; 2 Department of Anaesthesiology, Intensive Care, Emergency Medicine and Pain Therapy, Ziekenhuis Oost-Limburg, Genk, Belgium; 3 Department of Cardiology, Ziekenhuis Oost-Limburg, Genk, Belgium

**Keywords:** Cardiac arrest, Prognostication, Neuromonitoring, Targeted temperature management

## Abstract

**Background:**

Prognostication in out-of-hospital cardiac arrest (OHCA) survivors is often difficult. Recent studies have shown the predictive ability of bispectral index (BIS) monitoring to assist with early neuroprognostication. The aim of this study was to investigate whether characteristics of BIS values equal to zero (BIS 0) (i.e. duration and/or uni- versus bilateral presence) instead of simply their occurrence are better indicators for poor neurological outcome after OHCA by aiming at a specificity of 100%.

**Methods:**

Between 2011 and 2015, all successfully resuscitated OHCA patients were treated with targeted temperature management (TTM) at 33 °C for 24 hours followed by rewarming over 12 hours (0.3 °C/h). In total, BIS values were registered in 77 OHCA patients. The occurrence of unilateral (BIS 0 at one hemisphere) and bilateral (BIS 0 at both hemispheres) BIS 0 values as well as their total duration were calculated. Receiver operating characteristic (ROC) curves were constructed using the total duration with BIS 0 values calculated from the initiation of TTM onwards to determine poor neurological outcome.

**Results:**

In 30 of 77 OHCA patients (39%), at least one BIS 0 value occurred during the first 48 hours after admission. Of these 30 patients, six (20%) had a good (cerebral performance category (CPC) 1–2) and 24 (80%) a poor neurological outcome (CPC3–5) at 180 days post-CA. Within these 30 patients, the incidence of bilateral BIS 0 values was higher in patients with poor neurological outcome (CPC1–2: 2 (33%) vs. CPC3–5: 19 (79%); *p* = 0.028). The presence of a BIS 0 value predicted poor neurological outcome with a sensitivity of 62% and specificity of 84% (AUC: 0.729; *p* = 0.001). With a ROC analysis, a total duration of 30,3 minutes with BIS 0 values calculated over the first 48 hours predicted poor neurological outcome with a sensitivity of 63% and specificity of 100% (AUC: 0.861; *p* = 0.007).

**Conclusions:**

This study shows that a prolonged duration with (bilateral) BIS 0 values serves as a better outcome predictor after OHCA as compared to a single observation.

## Background

Despite improvements in cardiopulmonary resuscitation and intensive care treatment including targeted temperature management (TTM), hypoxic-ischaemic brain injury remains the predominant cause of death in out-of-hospital cardiac arrest (OHCA) patients admitted to the intensive care unit [1–3]. Early and reliable identification of patients with no prospect of favourable outcome avoids futile and expensive treatment prolongation and would be of value informing relatives. Currently, a multimodal strategy is being recommended for reliable neuroprognostication, encompassing modalities such as electro-encephalography (EEG), somatosensory evoked potentials (SSEP) and magnetic resonance imaging [4–7]. Still, most of these robust outcome predictors are labour-intensive, expensive, not continuous and above all require trained specialists for correct interpretation. Recently, the prognostic performance of simple bispectral index (BIS) monitoring has been investigated thoroughly in the post-cardiac arrest (CA) setting [8–15]. This monitoring option, originally designed to monitor the degree of awareness during anaesthesia, converts raw sampled frontal EEG signals into a simple and real-time BIS index that ranges from 0 (iso-electric EEG) to 100 (normal electrical activity in awake subjects). The presence of BIS values equal to zero (BIS 0) during TTM at 33 °C, equivalent to flat or low-voltage EEG, has been associated with poor neurological outcome [8]. However, as it has been shown that the presence of BIS 0 values does not reach a specificity of 100% on its own, it was recommended not to use the presence of low-voltage EEG or BIS 0 values on its own to predict poor outcome after OHCA [16]. We investigated whether characteristics of BIS 0 values (duration and/or uni- versus bilateral presence) during the first 48 hours after admission instead of simply their occurrence were indicative for poor neurological outcome after OHCA aiming at a specificity of 100%.

## Methods

In this prospective, observational study, all adult comatose survivors after OHCA with a presumed cardiac origin admitted to the coronary care unit (CCU) of Ziekenhuis Oost-Limburg (Genk, Belgium) were consecutively included between March 2011 and May 2015. Ethical approval was obtained before study onset (CME11/066) and written informed consent was obtained from the patient’s next of kin.

The institutional post-resuscitation protocol has been described previously [17, 18]. In summary, TTM at 33 °C was initiated immediately after admission to the emergency department by administering cold fluids intravenously (4 °C, 30 ml/kg). After admission to the CCU, TTM at 33 °C was further induced and maintained for 24 hours using either a surface-cooling (ArcticGel™ pads, Artic Sun System® 5000, Medivance, Louisville, CO, USA) or endovascular cooling system (Icy-cathether, CoolGard® 3000; Alsius, Irvine, CA, USA). Both systems were equipped with a feedback loop system to control target temperature using an oesophageal temperature probe. Twenty-four hours after CCU admission, patients were actively rewarmed towards a core temperature of 36.6 °C at a rate of 0.3 °C per hour. All patients were intubated, mechanically ventilated and sedation was induced and maintained by administering propofol, midazolam and remifentanil intravenously. Within the period of TTM, doses of sedative drugs were titrated to obtain values between –3 and –5 on the Richmond Agitation-Sedation Scale. According to the guidelines, cisatracurium was only administered in case of shivering [19]. EEGs were carried out on clinical indication and epileptic activity was treated with anti-epileptic drugs. Patients were extubated once their neurological, hemodynamic and respiratory status was recovered sufficiently.

In patients remaining comatose despite complete cessation of sedation, full supportive treatment was continued until at least 72 hours after rewarming. As such, withdrawal of life-sustaining therapy was never performed before this time point. In accordance with recommendations of international guidelines, signs of brain death (i.e. absent pupillary and corneal reflexes), refractory seizures and the bilateral absence of the N20 component of the SSEPs were taken into account for the decision to withdraw life support [20].

BIS VISTA™ monitoring with a six-electrode frontotemporal bilateral sensor was initiated after CCU admission (Aspect Medical Systems, Inc. Norwood, MA, USA). BIS values were stored per second during the first 48 hours, resulting in a maximum of 172,800 unilateral data points per patient. In addition, the signal quality index (SQI) and electromyographic (EMG) power were recorded continuously. The SQI refers to the signal accuracy where values above 80% are considered as reliable. The EMG power, (measured as dB), describes the electromyographic content of the EEG signal. The BIS VISTA™ device displays EMG bars according to the level of EMG noise where absence of EMG bars is indicative for EMG noise below 30 dB (i.e. a good signal quality) and presence of EMG bars means increasing EMG noise (1 bar: 30–38 dB up to 4 bars: >55 dB). Although physicians were not blinded to values displayed on the BIS monitor, decisions to withdraw life support or limit care were never based on the observed BIS values.

Outcome was assessed at 180 days post-CA using the Cerebral Performance Category (CPC) scale [21]. According to the scale classification, CPC1 is indicative for good cerebral performance; CPC2 implies a moderate disability with sufficient cerebral functioning for independent daily-life activity; CPC3 indicates severe neurological sequelae; CPC4 implies coma or vegetative state and CPC5 stands for death. In this study, a CPC1–2 and CPC3–5 was considered as good and poor neurological outcome, respectively.

Statistical analysis was performed using SPSS Version 24.0 (IBM Corp., Armonk, NY, USA). Equal distribution was tested by means of a Kolmogorov-Smirnov test. Depending on normality, categorical data were compared between patients with a good and poor neurological outcome using a Fisher exact or Chi-square test while unpaired *t* tests or Mann-Whitney *U* tests were used to compare continuous data. All data are presented as median (interquartile range (IQR)). Raw sampled BIS data were synchronized with the time TTM at 33 °C was initiated. The occurrence of unilateral (BIS 0 at left or right hemisphere) and bilateral (BIS 0 at both hemispheres) BIS 0 values was calculated together with their total duration within the first 48 hours from the initiation of TTM at 33 °C onwards. The concomitant EMG power and SQI values at the time BIS 0 values were present were analysed as well. The total duration with any BIS 0 value was calculated within the first 12 and 48 hours after TTM was initiated. Afterwards, receiver operating characteristic (ROC) curves were constructed using the total duration with any BIS 0 value (within the first 12 or 48 hours) to determine poor neurological outcome aiming at a specificity of 100%. *P* values < 0.05 were considered as significant.

## Results

**Fig. 1:**
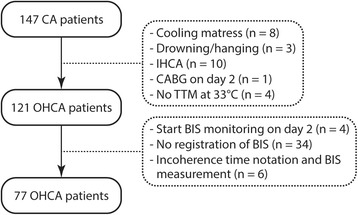
Flowchart of enrolled study patients. *CABG* coronary-artery bypass graft surgery, *IHCA* in-hospital cardiac arrest, *OHCA* out-of-hospital cardiac arrest, *TTM* targeted temperature management

One hundred and twenty-one eligible OHCA patients were prospectively enrolled between March 2011 and May 2015. Data of 44 patients were excluded from further analysis due to the following reasons: no registration of BIS values (n = 34), start BIS monitoring after day 2 (n = 4) and incoherence between time notation and start BIS measurement (n = 6). In total, 77 successfully resuscitated OHCA survivors with a cardiac cause of origin were prospectively included (Fig. 1).

**Fig. 2:**
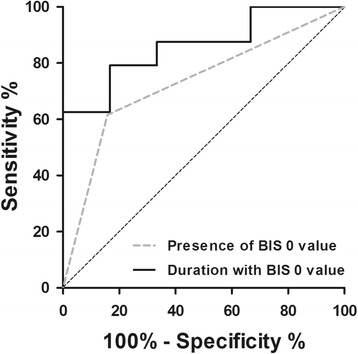
ROC curves of the presence and duration with BIS 0 values. The presence of a BIS 0 value predicted poor neurological outcome with a sensitivity of 62% (95% CI: 45–7) and specificity of 84% (95% CI: 45–77) (AUC: 0.729 (0.614–0.844)). The total duration with any BIS 0 values was calculated within the first 48 hours after TTM was initiated. A duration of 1820 seconds was associated with poor neurological outcome with a sensitivity of 62% (95% CI: 41–81) and specificity of 100% (95% CI: 54–100) (AUC: 0.861 (0.719–1.000))

Forty-seven out of these 77 patients (61%) never experienced a BIS value of 0 during the first 48 hours after CCU admission of whom 15 had a poor (32%) and 32 (68%) a good neurological outcome at 180 days post-CA. In contrast, at least one BIS 0 value was observed during the first 48 hours after CCU admission in 30 out of 77 patients (39%). At the moment BIS 0 values were recorded, mean SQI was 98 ± 1% and mean EMG was 26 ± 3 dB, indicating an adequate signal quality with insignificant interference. Of these 30 patients, six (20%) had a good and 24 (80%) a poor neurological outcome at 180 days post-CA. Baseline characteristics of these 30 patients are summarized in Table 1. Also, the incidence of bilateral BIS 0 values was higher in patients with a poor neurological outcome (CPC1–2: 2/6 (33%) vs. CPC3–5: 19/24 (79%); *p* = 0.028). The presence of a BIS 0 value within the first 48 hours predicted poor neurological outcome with a sensitivity of 62% (95% CI: 45–76) and specificity of 84% (95% CI: 68–93) (AUC: 0.729 (0.614–0.844); *p* = 0.001; Fig. 2). This corresponded to a positive predictive value (PPV) of 80% (95% CI: 61–92), a negative predictive value (NPV) of 68% (95% CI: 53–80) and false positive ratio (FPR) of 20% (95% CI: 8–39).

**Table 1:** Demographics

	Good neurological outcome (*N* = 6)	Poor neurological outcome (*N* = 24)	*P* value
Demographics			
Age	66 (49–70)	67 (56–79)	0.27
Male	5 (83)	21 (88)	0.79
Initial rhythm			0.60
Shockable	4 (67)	17 (77)	
Non-shockable	2 (33)	5 (22)	
Witnessed arrest	6 (100)	20 (87)	0.35
Time to target temperature (min)	174 (90–294)	147 (101–229)	0.85
Time emergency call – ROSC (min)	38 (30–38)	35 (22–39)	0.48
Neuron-specific enolase (μg/l)			
Hour 24	28 (21–57)	86 (55–110)	**0.006**
Hour 48	48 (15–62)	156 (71–278)	**0.003**
Electro-encephalography			
Burst suppression	1 (16)	11(46)	0.36
Status epilepticus	0 (0)	10(42)	0.07
Use of sedatives and neuromuscular blockage			
Max. dose propofol (mg/kg/hour)	3.30 (1.55–5.78)	2.20 (1.78–2.53)	0.14
Max. dose remifentanil (μg/kg/min)	0.12 (0.07–0.13)	0.10 (0.08–0.13)	0.96
Neuromuscular blockage use	3 (50)	15 (63)	0.66

Values are shown as median with 25 and 75 percentile and n (%)

Significant values are indicated in bold

*ROSC* return of spontaneous circulation

The median recording time in patients with a good and poor neurological outcome was 2880 (IQR: 2728–2880) and 2880 (IQR: 2521–2880) minutes, respectively (*p* = 0.631). The median duration with unilateral BIS 0 values during the first 48 hours was 3 (IQR: 0–21) and 17 (IQR: 8 – 49) minutes in patients with a good and poor neurological outcome, respectively (*p* = 0.052). The median duration with bilateral BIS 0 values was higher in patients with a poor neurological outcome (CPC1–2: 0 (IQR: 0–3) vs. CPC3–5: 13 minutes (IQR: 0–219); *p* = 0.016). Within the first 48 hours, the total median duration with any BIS 0 value was 8 (IQR: 1–21) and 49 (IQR: 23–378) minutes in patients with a good and poor neurological outcome, respectively (*p* = 0.007). By means of a ROC analysis, a total duration of 1820 seconds (i.e. 30, 33 minutes) with BIS 0 values calculated over the first 48 hours predicted poor neurological outcome with a sensitivity of 63% (95% CI: 41–81) and specificity of 100% (95% CI: 54–100) (AUC: 0.861 (0.719–1.000); *p* = 0.007; Fig. 2). With this cut-off value, a PPV of 100% (95% CI: 75–100), a NPV of 40% (95% CI: 17–67) and FPR of 0% (95% CI: 0–25) was calculated. An additional ROC analysis was performed using the total duration with BIS 0 values calculated over the first 12 hours to assess the possibility to use the BIS monitor as a triage method after OHCA. A total duration of 1810 seconds (30.17 minutes) with BIS 0 values calculated over the first 12 hours predicted poor neurological outcome with a sensitivity of 57% (95% CI: 34–77) and specificity of 100% (95% CI: 54–100) (AUC: 0.855 (0.703–1.000); *p* = 0.008).

**Fig. 3:**
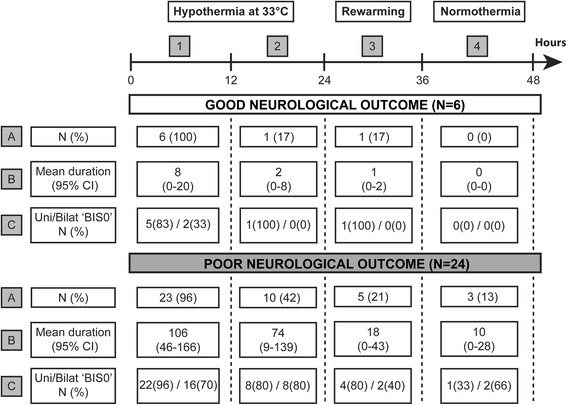
Overview of the characteristics of BIS 0 values within specific time periods. In total, six patients with a good and 24 with a poor neurological outcome experienced at least one BIS 0 value within the first 48 hours following CCU admission. After subdividing this 48-hour time period into four equal time frames (denoted as *1–4* in the figure), the proportion of patients (**a**) with their respective mean duration of BIS 0 in minutes (**b**) was calculated per phase for both outcome groups. Additionally, the percentage of patients experiencing unilateral (BIS 0 at one hemisphere) or bilateral (BIS 0 at both hemispheres) BIS 0 values (**c**) is represented within each phase as well

The first 48 hours after the induction of TTM at 33 °C can be subdivided in (1) a hypothermic phase (0–24 h), (2) a rewarming phase (24–36 h) and (3) the normothermia phase (36–48 h). BIS 0 values were observed during hypothermia in all six patients with a good and in 23 out of 24 patients (96%) with a poor neurological outcome (Fig. 3). Seven patients had BIS 0 values during the rewarming phase of whom one (16%) with a good and five (22%) with a poor neurological outcome. Three patients (13%) with poor neurological outcome experienced BIS 0 values during normothermia in contrast to none of the patients with a good neurological outcome, corresponding to a sensitivity of 13% (95% CI: 3–33) and specificity of 100% (95% CI: 52–100).

## Discussion

This study shows that the duration with BIS 0 values rather than a single observation serves as a better outcome predictor after OHCA. Thirty OHCA patients (38%) experienced a BIS 0 value during TTM. Still, six of them attained a good neurological outcome. In contrast, a total duration with BIS 0 values beyond half an hour was uniformly associated with poor neurological outcome at 180 days post-CA. Furthermore, bilateral BIS 0 values seem to be of better prognostic value as compared to unilateral BIS 0 values.

To date, early neuroprognostication in OHCA survivors remains challenging. Current guidelines now recommend the use of a multimodal neuroprognostication algorithm containing prognostic tools such as EEG, SSEP and neuroimaging. Although these robust predictors do reach a high specificity on their own, they require trained physicians for correct interpretation and are often considered as time-consuming and expensive [4–7]. Due to its simple and non-invasive nature, BIS monitoring has been investigated for its potential to assist with early neuroprognostication. The phenomenon that OHCA patients displaying lower BIS values are prone to a worse neurological outcome has gained acceptance in recent years [8–14]. Several studies reported that the presence of BIS 0 values at any point up to 24 hours could be considered as an early indicator for poor outcome (specificity: 100%) [8–10]. This was mitigated by a subsequent study in which BIS 0 values predicted poor neurological outcome with a specificity of only 90% [11]. This is consistent with our results in which the presence of a BIS 0 value corresponded with a specificity of 84%. In total, six OHCA patients experienced BIS 0 values and attained a good neurological outcome at 180 days post-CA. However, this is the first study showing that the duration of periods with BIS 0 values rather than a single observation serves as a better outcome predictor after OHCA. Within the first 48 hours after TTM was initiated, all patients who experienced BIS 0 values exceeding half an hour had a poor neurological outcome (sensitivity: 63% and specificity: 100%). Given the described confounding impact of neuromuscular activity on the BIS index, we cannot exclude the possibility that the calculated sensitivity would have been even higher if neuromuscular blockers (NMB) would have been administered continuously [9]. Interestingly, a nearly similar predictive accuracy was reached if only the first 12 hours were taken into account. This is in line with Stammet et al. which recently demonstrated that a mean BIS below 2.4 calculated over the first 6.5 hours was a certain predictor for poor outcome [14]. In this way, our findings contribute to the concept that a prolonged duration with low BIS values in the early hours after cardiac arrest could be used to guide early post-cardiac arrest triage [9, 12–14, 22].

Another remarkable observation was the lower incidence and shorter duration with bilateral BIS 0 values in the six patients with a good neurological outcome. Since we are the first to report this finding, we can only speculate that most patients with unilateral BIS 0 values might have had sufficient cerebral reserve allowing the contralateral hemisphere to recover from small time periods of cortical inactivity. Another possibility, on the other hand, is that the incidence with bilateral BIS 0 values would have been higher with the continuous administration of NMB. Hence, future studies using bilateral BIS monitoring concomitantly with continuous full EEG are required to confirm these preliminary results and need to elucidate the impact of NMB on the uni- or bilateral appearance of a BIS 0 value.

Especially during hypothermia, BIS values as low as zero can be observed in patients with good neurological outcome. It has been shown that cerebral ischemia induces an acute failure of synaptic transmission within the first minute following circulatory arrest resulting in a flat EEG [23, 24]. Therefore, our results confirmed that iso-electric EEG patterns or BIS values of 0 are not uncommon in the early hours after cardiac arrest, but do not preclude full recovery of brain function. On the other hand, 25% of patients with poor neurological outcome experienced BIS 0 values after the hypothermic phase was ended. This is consistent with published data where the presence of an initial flat EEG during TTM at 33 °C was shown to be of no prognostic value and more importantly, an EEG pattern evolving from flat towards continuous EEG lines was predictive for good neurological outcome [25–30]. In analogy with these results, others demonstrated that the presence of iso-electric or low-voltage EEGs 24 hours after resuscitation, but not earlier, was a strong indicator of poor neurological outcome (specificity: 100%) [6, 31–33]. As such, our results strengthen the hypothesis that persisting suppression of cortical activity after the end of TTM at 33 °C is associated with an increased mortality risk. Nevertheless, one should be aware that BIS indices remain an automated calculation of frontal EEG activity and, unlike conventional EEG, are not reliable to use for diagnostic purposes [30, 34].

This study has several limitations. First, BIS values were not blinded because signal quality assessments required visual confirmation. Despite this, BIS 0 values were never used in the decision to withdraw life support. In fact, treating physicians were cardiologists who were most often not familiar with the use of BIS monitoring. Second, previous studies showed that hypothermia lowers the BIS value [35, 36]. However, it was unlikely TTM affected our results as the applied temperature regimen was uniform in all patients and the time to target temperature was not different between both outcome groups. Still, the potency of the duration with BIS 0 values to predict poor neurological outcome in patients treated with TTM at 36 °C remains unanswered, as TTM at 33 °C was applied in our patient cohort. Third, the exact time to return of spontaneous circulation (ROSC) and the specific time span between ROSC and the initiation of BIS monitoring were unknown, which could be considered as a limitation. In order to have a similar starting point for all patients, it was therefore decided to synchronize the raw sampled BIS data with the time TTM at 33 °C was initiated. Fourth, the total sample size was relatively small although 39% of all patients experienced BIS 0 values. Due to the limited sample size, it is not possible to exclude that in a larger database, OHCA patients presenting with BIS 0 values exceeding half an hour, might attain a good neurological outcome. Therefore, this (preliminary) study should be considered as a hypothesis-generating one which raised some interesting thoughts that should be confirmed in future large-scale trials. In addition, these studies should focus on the contribution of the duration with BIS 0 values to the standardized neuroprognostication algorithm recommended by current guidelines.

## Conclusions

This study demonstrated at first that a prolonged duration with BIS 0 values serves as a better outcome predictor after OHCA as compared to a single observation. Although less specific, isolated BIS 0 values remained associated with a poor neurological outcome. Despite these promising results, the validity of this prognostic parameter in clinical practice remains inconclusive due to the small number of patients studied.

## Abbreviations

BIS: Bispectral index
BIS 0: BIS values equal to zero
CA: Cardiac arrest
CCU: Coronary care unit
CPC: Cerebral performance category
EEG: Electro-encephalography
EMG: Electromyographic power
FPR: False-positive range
IQR: Interquartile range
NMB: Neuromuscular blocker
NPV: Negative predictive value
OHCA: Out-of-hospital cardiac arrest
PPV: Positive predictive value
ROC: Receiver operating characteristic
ROSC: Return of spontaneous circulation
SQI: Signal quality index
SSEP: Somatosensory evoked potential
TTM: Targeted temperature management
